# Evaluation of treatment efficacy after switching to dolutegravir-lamivudine dual therapy in people living with HIV

**DOI:** 10.4314/ahs.v22i3.46

**Published:** 2022-09

**Authors:** Pınar Ergen, Begüm Bektas, Özlem Aydın, Havva Keskin, Ayşe Canan Üçışık, Fatma Yılmaz Karadağ, Yasemin Cağ

**Affiliations:** 1 Department of Infectious Diseases and Clinical Microbiology, Istanbul Medeniyet University Göztepe Training and Research Hospital, Istanbul, Turkey; 2 Department of Internal Medicine, Istanbul Medeniyet University Göztepe Training and Research Hospital, Istanbul, Turkey; 3 Department of Infectious Diseases and Clinical Microbiology, Istanbul Medeniyet University Faculty of Medicine, Istanbul, Turkey

**Keywords:** dolutegravir, lamivudine, dual, antiretroviral therapy, efficacy

## Abstract

**Background:**

People living with HIV need to use antiretroviral therapy throughout their lives.

**Objectives:**

Studies on the efficacy and safety of dual therapy are limited in Turkey. We sought to evaluate the treatment efficacy and side effects among patients who were given a combination of dolutegravir (DTG) and lamivudine (3TC) as a maintenance therapy.

**Methods:**

This retrospective, single-centre study included individuals with viral suppression who were older than 18 years of age, living with HIV, switched from a combination antiretroviral therapy regimen to DTG-3TC dual therapy, and followed up for at least 6 months.

**Results:**

The study included 63 patients living with HIV. The median age was 42 years (interquartile range (IQR): 36–51 years). The median follow-up under the DTG-3TC regimen was 10.4 months (7.1–16.0 months). In the course of dual therapy, no patients developed any serious adverse effects that would necessitate a therapy switch, but virological blips were seen in two patients. Two patients lost their lives, with one dying from suicide and one dying from respiratory failure associated with the underlying chronic obstructive pulmonary disease.

**Conclusion:**

The DTG-3TC dual-therapy regimen is a promising and effective therapy that can be used as a treatment of choice for eligible patients.

## Introduction

The treatment of HIV has undergone substantial changes and improvements since the first introduction of zidovudine (ZDV) monotherapy in 1987, followed by combination antiretroviral therapy (cART)/high-activity antiretroviral therapy (HAART) in 1996. Combination therapy is still a current treatment of choice and is based on the use of 3 drugs, of which at least 2 are from different groups. One example involves two nucleos(t)ide analogue reverse transcriptase inhibitors (N(t)RTIs) and one of the following: a non-nucleoside reverse transcriptase inhibitor (NNRTI), amplified protease inhibitor (PI), integrase inhibitor, fusion inhibitor, or attachment inhibitor. Although a complete cure cannot be achieved with current ART regimens, the aim is to reduce HIV-associated morbidity and mortality, to lengthen the life span of people living with HIV (PLWH), and to prevent HIV transmission^1^–^3^.

Attempts to achieve treatment goals have often been complicated by adverse drug effects, drug-drug interactions, and costs. This issue, motivated the development of different drug regimens, particularly dual therapies. Many different studies have been conducted in different countries regarding dual treatment regimens based on PI such as atazanavir (ATV), lopinavir (LOP), darunavir (DRV) and Integrase inhibitors such as Dolutegravir (DTG), raltegravir (RAL)^4^–^6^. DTG - Rilpivirine (RPV), DTG - lamivudine (3TC), DTG - DRV, DTG - ATV regimens have been tried in DTG-based treatments^7^–^9^. Thus, the goal of the present study was to evaluate the treatment efficacy of DTG-3TC as a dual regimen and adverse effects that might necessitate a medication switch.

## Materials and Methods

We retrospectively evaluated patients who had been under outpatient follow-up with a diagnosis of HIV infection at the Infectious Disease and Clinical Microbiology Clinic of Istanbul Medeniyet University from January 2016 and April 2021. The study protocol was approved by the Ethics Committee of Istanbul Medeniyet University, Goztepe Training Hospital and the study was conducted in accordance with the principles of the Helsinki Declaration (2020/0123).

### Patients

The study included non-naive patients who had no viral failure, for whom the triple ART regimen had been switched to a DTG-3TC maintenance dual regimen due to drug side effects (bone toxicity, renal toxicity, hyperlipidemia, drug hypersensitivity, etc.), outdated current treatment, drug-drug interaction, or simplification. Of the 1006 patients who presented to the outpatient clinic of infectious diseases, 74 were identified as receiving dual treatment, but 11 patients were excluded from the study because 8 of them had received a dual treatment for less than 6 months, and 3 of them had missing data. The inclusion criteria were as follows: living with HIV, age 18 years or older, absence of pregnancy, receipt of any triple ART regimen prior to the dual regimen, an HIV RNA level of less than 100 IU/ml or an undetectable level for at least 6 months, and receipt of the DTG-3TC dual regimen for at least 6 months. All patients had a CD4+T lymphocyte count of more than 200 cells/mm3 at the time of switching. The cARTs that were administered before the dual treatment were recorded.

### Definitions^10^,^11^

Viral suppression refers to a decrease in the plasma HIVRNA level to below the detection threshold of the test used, remaining constant at that level, being less than 50 copies/ml, or being at an undetectable level. The test-dependent limit used in the current study was accepted as less than 100 IU/mL.

Viral failure refers to a level of HIV-RNA of more than 200 copies/ml at week 24 and the inability to maintain the suppression of viral replication.

Virological blip refers to the detection of a transient increase in HIV RNA (50 to 400 copies/ml) after viral suppression is achieved.

### Virological and biochemical analysis

Due to its retrospective design, decisions to switch the ART were derived from patients' records with clinicians' decisions. HIV RNA levels (100 to 100,000,000 IU/mL) were tested with a real-time PCR method (ARTUS QIAGEN HI Virus-1 RG RT-PCR), and the CD4 T lymphocyte count was tested with a standard flow cytometry method (FACS calibur, Becton Dickinson). Total cholesterol and triglyceride levels were evaluated after a 12-hour fast. Levels of total cholesterol (upper limit 200 mg/dL) and triglyceride (upper limit 150 mg/dL) were assessed as normal or high, and co-morbidities were taken into account. Renal functions were evaluated with the estimated glomerular filtration rate (e-GFR), and GFR ≥60 was considered normal.

### Statistical analysis

The distribution characteristics of the data as well as distribution characteristics of the differences between the dependent variables were analyzed with both normality tests and graphs (histogram graph, normal Q-Q plot and detrended normal Q-Q plot graphs). For descriptive statistics, mean standard deviation, median (interquartile range-IQR), or number and frequency values were used, when appropriate.

For continuous data, the Wilcoxon test or paired-sample T-test was used for dependent variables and the Mann-Whitney U-test or student's T-test was used for independent variables. Data were processed using IBM SPSS statistics (version 25.0 for Windows, Chicago, IL, USA) and a p-value of less than 0.05 was considered significant.

## Results

The current study included 63 patients (62 men, 1 woman) with a median age of 42 years (interquartile range (IQR): 36 to 51 years). The youngest patient was 23 years old, and the oldest was 77 years old. 51.61% (n=32) of men had sex with other men (MSM). The earliest diagnosis was made in 2003, and the most recent was made in 2020. The average time of diagnosis was 5 years (4 to 7 years). There were 15 patients (23.8%) who had co-morbidities (64.3% of them were 50 years of age or older), of which the most common was hypertension. Table 1 shows the descriptive characteristics of the patients and other co-morbidities.

**Table 1:** Distribution of Descriptive Characteristics of the Patients

	All cases (n=63)

	n (%) or median (IQR)
**Age (years)**	42 (36–51)
**Male/Female**	62/1 (98.4/1.6)
**HIV Risk factor**	
**MSM**	31 (49.2)
**Heterosexual** *	32 (50.8)
**Comorbidities**	15 (23.8)
Hypertension	10 (15.9)
Congestive heart disease	5 (7.9)
Chronic obstructive pulmonary disease	4 (6.3)
Psychiatric disorder	5(8.0)
Chronic renal failure	2 (3.2)
Diabetes mellitus	4 (6.3)
Rheumatological disease	2 (3.2)
Hypothyroidism	1 (1.6)
Hepatitis B virus co-infection	1 (1.6)
**The time to HIV diagnosis (years)**	5 (4–7)
**The duration of treatment (months)**	55.8 (43.5–78.3)
The duration of triple treatment	41.4 (30.4–59.1)
The duration of dual treatment	10.4 (7.1–16.0)

* Of patients, only one was woman

The median treatment durations of patients with viral suppression on triple therapy was 41.4 months (IQR: 30.4–59.1 months). The duration of the treatment was 10.4 months (7.1–16.0 months) after the switch, and the duration of total ART was 55.8 months (43.5–78.3 months). Table 2 shows all combinations and distributions of the triple ART regimen administered before dual therapy. The most common combination regimen was 2NRTI-INSTI (n:51), followed by NRTI-PI (n:17). The least administered one was NRTI-NNRTI (n:6). Among those who received 2NRTI-INSTI, the most common combination regimen was TDF-FTC-DTG (54%). 63 patients were given 74 triple classical therapy, as in 11 patients, triple classical therapy was switched to another triple combination therapy before the dual therapy.

**Table 2:** Antiretroviral therapies used by patients before dual therapy and their distributions

	n (%)
**2 NRTI-PI/r (n=17)**	
• TDF-FTC-LOP	14 (22.2)
• ZDV-3TC-IND	1 (1.6)
• ZDV-3TC-LOP	2 (3.2)
**2NRTI-NNRTI (n=6)**	
• TDF-FTC-EFV	6 (9.5)
**2NRTI-INSTI (n=51)**	
• TDF-FTC-DTG	34 (54.0)
• TDF-FTC-ELV	3 (4.8)
• TDF-FTC-RAL	7 (11.1)
• TAF-FTC-ELV	5 (7.9)
• ABC-3TC-DTG	2 (3.2)

Abbreviations(t)RTI: tenofovir disoproxil fumarate(TDF), tenofovir alafenamide (TAF) emtricitabine (FT C) lamivudine (3TC) abacavir (ABC) zidovudine ( ZDV); NNRTI: efavirenz (EFV); INSTI raltegravir (RAL), elvitegravir ( ELV), dolutegravir (DTG); PI: lopinavir( LOP), indinavir( IND)

The main reason for switching to dual therapy was the detection of decreased bone mineral density (56.7%), which led clinicians to switch to dual therapy from triple therapy. Three patients were switched to dual therapy due to the development of hyperlipidemia during the treatment, and 13 patients were switched due to the development of renal dysfunction. Table 3 shows the reasons for switching to dual therapy and their frequencies.

**Table 3:** Reasons for and frequencies of switching to dual therapy

Reasons for switch	n (%) *
Osteopenia	32 (43.2)
Osteoporosis	10 (13.5)
Renal dysfunction	13 (17.6)
Drug hypersensitivity	1 (1.4)
Adverse gastrointestinal effects	2 (2.7)
Drug-drug interactions	3 (4.1)
Hyperlipidemia	3 (4.1)
Simplification	8 (10.8)
Outdated cART	2 (2.7)

* While the total number of cases was 63, the number of drug switch reasons was 74, since drug(s) were switched for one or more reasons. Data obtained during the triple treatment and after switching to dual therapy were compared.

There was no significant difference in CD4+T lymphocyte counts during the triple therapy and dual therapy (p = 0.154), and there was no significant difference in HIV RNAs. Two patients were found to have virological blips during dual therapy (215 IU/mL and 153 IU/mL). There were no significant differences in total cholesterol, triglyceride, and e-GFR levels between both treatments (p = 0.801, 0.819, and 0.538, respectively; Table 4).

**Table 4:** Comparison between triple ART and dual ART

	Triple ART (n=63)	Dual ART (n=63)	P value

	mean±SD or median (IQR)		
CD4+T cell count (cells/µl)	723 (479–970)	746 (590–1079)	0,154*
Total cholesterol (mg/dL)	200±48	203±47	0,801**
Triglyceride (mg/dL)	124 (86–178)	117 (87–185)	0,819*
e-GFR (ml/dk/1.73m^2^)	87±20	86±21	0,538**

* Wilcoxon test

** Paired-Sample T test

**Figure 1:**
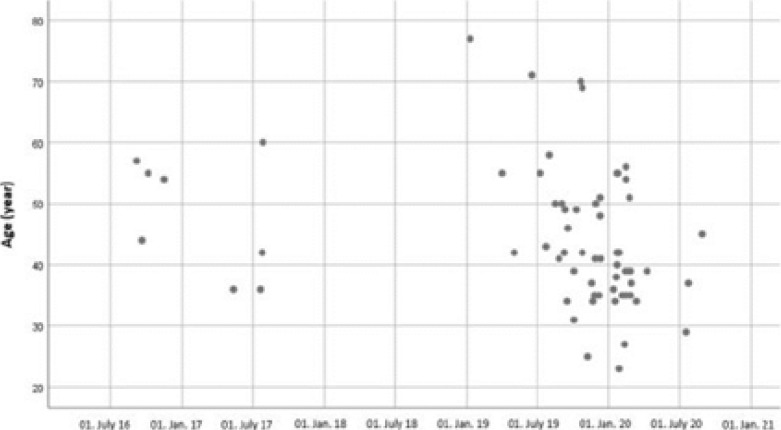
Date of switching from triple ART to dual therapy and the relationship between the age of the patients

We also evaluated whether age was a factor in the decision to switch from triple ART to dual ART and found that it had no effect. Figure 1 shows the relationship between the age of the patients and the date of switching from triple therapy to dual ART. As of 2019, an increase was noted in the frequency of switching to dual treatment.

## Discussion

The results suggest that virological suppression was maintained, there was no change in CD4+T lymphocyte counts among patients who were switched to dual therapy after triple therapy, and dual therapy was well tolerated. With the introduction of combined antiretroviral therapy, HIV infection has changed from a fatal disease to a chronic disease, resulting in a near-normal life expectancy among patients treated successfully^12^. The goal is to suppress viral load, boost the immune system, protect patients from opportunistic infections, and prevent transmission to other people. For this purpose, patients need to use ART regimens throughout their lives.

Despite ARTs increasing life expectancy of HIV-infected individuals, potential comorbidities associated with these medications have been a concern, including cardiovascular diseases, kidney diseases, bone pathologies, and diabetes^13^. In the current study, 15 patients were identified to have comorbidities, and 64.3% of them were 50 years of age or older. Hypertension and congestive heart failure were the most common comorbidities, followed by chronic obstructive pulmonary disease, psychiatric disorders, chronic renal failure, diabetes mellitus, rheumatological diseases, hypothyroidism, and hepatitis B virus co-infection. ART regimens are likely to contribute to these comorbidities and many others throughout the lives of patients^14^–^17^.

cART has been recommended as the primary therapy by previous guidelines. It is currently administered and include a third agent (NNNRTI/PI/INSTI) with a potent dual-NRTI backbone^18^,^19^. Current guidelines also recommend the combination of dolutegravir and lamivudine as a first-line regimen^10^,^20^. Dual therapy seems to a preferred treatment option in several aspects: reducing drug toxicity that may develop over time in eligible patients, preventing drug-drug interactions, improving adherence, and reducing costs. We hypothesized that dual therapy could be beneficial in reducing drug-related toxicity and interactions in patients with comorbidities.

There are many studies on DTG-3TC dual therapy in different countries around the world, which cover both naive and patients who were switched from another therapy. In a study on naive patients, Cahn et al.^21^ showed that a virological success rate of 90% was maintained at 48 weeks of therapy. Similarly, Nyaku et al.^22^ reported a virological success rate of 85%, and Taiwo et al.^23^ reported a rate of 90% at 52 weeks.

Among patients who received a switched regimen, Maggiolo et al. 8 reported that a virological success of 100% was maintained at week 24, and Jolly et al.^24^ reported a virological success rate of 97% at week 48. In Turkey, however, not many large studies have been performed in this regard. Yagli Caglaik et al.^25^ and Karabay et al.^26^ reported no serious adverse effects in the follow-up of patients who received a DTG-3TC regimen. Sonmezer et al.^27^ showed that viral suppression was achieved at one month among naive patients, and viral suppression was maintained in those who were switched to dual therapy. The current study included 63 patients with a follow-up period of at least 6 months. Although our number of patients and follow-up period were limited, our study contributes to the literature as it reflects the experience of a centre in Turkey.

Lamivudine inhibits reverse transcriptase, thus terminating proviral DNA chain extension and preventing HIV replication competitively. It is a well-tolerated drug with high absorption after oral administration, as well as enhanced bioavailability. It can be taken with or without food, has a favorable safety profile, has no significant drug-drug interactions, and is less expensive.^28^ Dolutegravir is an integrase strand transfer inhibitor that inhibits viral DNA chain transfer. DTG is a potent antiviral that is taken once daily with or without food, has a high genetic barrier, and has few drug-drug interactions^29^–^30^. Both agents can be taken at the same time, but there is also a single tablet form of the drug (300 mg 3TC and 50 mg DTG), although it is not yet available in Turkey^31^.

The present study demonstrated that as of 2019, dual therapy has become a preferred regimen more often than in previous years. The reason for this may be that the guidelines were updated in 2019 and encouraged physicians to switch to dual therapy^10^,^32^. The current study found that nephropathy was the most significant reason for switching to dual therapy before 2019 (n=7/8), and the reason for the preference for DTG-3TC was probably the need for antiviral agents with reduced toxicity.

Throughout the study period, the need for switching therapy was independent of age and was mostly due to decreased bone mineral density (osteopenia-osteoporosis). In PLWH, the balance between osteoblastic and osteoclastic activity is impaired, and some ARTs are known to contribute to this condition. In a meta-analysis, Goh et al. reported that there was a 2-fold increased risk of osteopenia/osteoporosis in PLWH undergoing an ART regimen as compared with the control group^33^. Lara et al. reported that bone mineral changes were detected in 47.8% of the patients in their study of 92 PLWH^34^. Clinical studies show that the use of tenofovir in particular is associated with this condition^35^,^36^. The present study found that all patients who were switched to dual therapy due to osteopenia/osteoporosis had received tenofovir-based NRTI during their previous therapies. Nonetheless, bone mineral loss may not be entirely attributed to the use of tenofovir, and it should be kept in mind that an underlying disease is likely to play a part.

PLWH also have a risk of nephropathy, which may be caused by both the direct effects of the virus and the antivirals on the kidneys^37^. In addition to the use of ARTs such as tenofovir disoproxil fumarate, an increase in the incidence of comorbidities such as diabetes and hypertension may also be associated with risk^38^,^39^. Borghetti et al. showed that renal toxicity was the reason for switching to DTG-3TC treatment in 11.1% of cases, while Tenorio et al. reported a rate of 21.6%^40^,^41^. Renal dysfunction was noted as the second most common reason for switching drugs in our study. The physicians considered a change in ART regimen due to renal dysfunction in 13 patients, of whom 12 (92.3%) had been receiving tenofovir disoproxil fumarate as a backbone therapy.

Hyperlipidemia is also an unfavourable condition that is noted in the follow-up of HIV and needs to be managed. Tenorio et al^41^. showed hyperlipidemia as the reason for switching 33.8%. The use of PIs is frequently associated with hyperlipidemia^42^. In the present study, 3 patients switched therapies due to hyperlipidemia, and all of them had been receiving a combination therapy with PI (lopinavir/ritonavir).

The treatment of one of our patients was changed due to drug hypersensitivity. The patient presented with dyspnoea in the 4th week of ABC-DTG-3TC treatment. Consolidated areas of the lungs were detected in thorax CT, and infectious processes were ruled out. The complaints regressed after discontinuation of the patient's antiviral treatment. The patient had been receiving DTG-3TC therapy since June 27, 2017, and reported no complaints. ABC-dependent drug hypersensitivity has been reported in individuals who are positive for HLA 5701, and it can rarely be seen in negative patients. Stainsby et al. reported a rate of 1.3% or less for ABC-related hypersensitivity in their study on HLA 5701-negative patients^43^. Our patient's pre-treatment showed negative results on the HLA 5701 test.

Two patients switched ARTs due to persistent gastrointestinal adverse events, such as diarrhoea and indigestion, which improved after switching. Both patients were receiving PI (one patient lopinavir, the other indinavir), for which adverse events of the gastrointestinal system have been reported^44^.

A patient with a history of frequent hospitalizations for chronic obstructive pulmonary disease died from respiratory failure caused by exacerbation of the underlying disease while having a negative viral load. Another patient died from suicide. The patient had received a diagnosis of depression and had been receiving treatment for 4 years. Before switching to DTG-3TC therapy, the patient had been using cART containing DTG for 21 months and was HIV RNA negative until the last follow-up visit. Mental health problems are known to be frequent in PWLH, with depression being the most common psychiatric co-morbidity after substance misuse^45^. The patient had a diagnosis of depression before receiving ART and was under the follow-up of a physician with this diagnosis. It is therefore difficult to attribute his death to ART. The combination of DTG-3TC is not recommended as an antiretroviral treatment for HBsAg-positive patients20. Nevertheless, DTG-3TC treatment was preferred for one patient, providing that the patient assured the clinician that they would adhere to follow-up visits. At a 4 year follow-up for dual therapy, the patient was negative for HBV DNA.

During the follow-up period, no side effects that would necessitate a change in treatment were encountered in patients who received dual therapy. DTG-3TC treatment was found to be an effective alternative with fewer side effects, it was easy to use, and it might be cost effective. However, since the study lacked a control group and had a retrospective design, there are missing data. Disruptions in routine outpatient services due to the COVID-19 pandemic and patients not seeking care were also limitations of the study.

## Conclusion

The choice of ART in the treatment of PLWH needs to be specific to the individual. Factors such as co-morbidities, drug toxicities, patient adherence to medications, and drug-drug interactions should be taken into consideration. DTG-3TC dual treatment seems to be an effective and reliable option in the long run with increased adherence to therapy, making it an appropriate alternative for eligible patients.

## Approval

The study protocol was approved by the Ethics Committee of Istanbul Medeniyet University, Goztepe Training Hospital and the study was conducted in accordance with the principles of the Helsinki Declaration (2020/0123).
